# Therapeutic use of serious games in mental health: scoping review

**DOI:** 10.1192/bjo.2022.4

**Published:** 2022-02-02

**Authors:** Alice Dewhirst, Richard Laugharne, Rohit Shankar

**Affiliations:** University of Exeter Medical School, UK; Cornwall Intellectual Disability Equitable Research, University of Plymouth Medical School, UK; and Cornwall Partnership NHS Foundation Trust, UK; Cornwall Intellectual Disability Equitable Research, University of Plymouth Medical School, UK; and Cornwall Partnership NHS Foundation Trust, UK

**Keywords:** Serious games, depression, anxiety, schizophrenia, bipolar disorder

## Abstract

**Background:**

There has been an increase in the development and application of serious games to support management of mental ill health, but their full impact is unclear.

**Aims:**

Evaluation of the current evidence of acceptability and effectiveness of serious games in improving mental health disorders.

**Method:**

A PRISMA-guided scoping review was conducted, using a predefined criteria and a relevant word combination on three databases: EMBASE, Medline and PsycINFO. Each included study was examined for game format, study type, number of participants, basic demographics, disorder targeted, recruitment, setting, control conditions, duration and follow-up, study attrition, primary outcomes and their results. Each study was given a Grading of Recommendations, Assessment, Development and Evaluations rating for quality.

**Results:**

Fourteen out of 513 studies met the inclusion criteria. The serious games focused on symptoms of anxiety (*n* = 4), attention-deficit hyperactivity disorder (*n* = 3), depression (*n* = 2), schizophrenia (*n* = 2), alcohol use disorder (*n* = 2) and bipolar disorder (*n* = 1). There were multiple significant outcomes favouring serious games across conditions covered in the review. Study quality varied, with studies rated high (*n* = 3), moderate (*n* = 6), low (*n* = 3) and very low (*n* = 2).

**Conclusions:**

The available evidence suggests that serious games could be an effective format for an intervention to reduce mental health symptoms and improve outcomes of individuals. Better designed studies would further develop confidence in this area. This is a potential vehicle of change to deliver some of the much-needed psychiatric support to both economically developed and developing regions in a resource-utilitarian manner. Partnerships between the gaming industry, researchers and health services may benefit patients.

Mental illness is one of the biggest health burdens worldwide.^1^ It is prevalent among all age groups and early access to treatment can improve outcomes.^2^ In the UK, people can wait up to 18 weeks for a referral to consultant-led mental health services.^3^ In low- and middle-income countries, a high proportion of people do not receive any treatment, and the need for improved access to treatment is increasing.^4^ With high demand, health systems need to be able to adapt to cope with the influx of referrals.

## Digital interventions and gaming

E- interventions are already being used across healthcare and may be more accessible options. These can take various forms, such as text-based programmes, multimedia and interactive programmes that use emails or text messages, biofeedback programmes, virtual reality and serious games.^5^ The evidence for all of these is developing and has evolved to different extents.

## Serious games

Serious or applied games are games (usually video platform) devised to have a primary purpose than just as recreation.^6^ Industries like healthcare, education, engineering and defence use video games that can described as ‘serious’.^7^

Serious games are designed to educate, train, or change behaviour as they entertain the game players.^8^ Serious games have been used in numerous health settings and could be a new, accessible option for many patients in mental health services.^9,10^ A wide range of commercial games have potential in mental health disorders despite this not being their designed purpose.^11^ This potential could be further developed by integrating games with therapeutic elements, such as cognitive–behavioural therapy (CBT).^12^

This is a fast-moving field of endeavour. There have been good-quality systematic reviews of serious games in depression and mental health published between 2014 and 2017.^13–16^ They suggested that there were promising signs that serious games could be an effective treatment, but the research was at an early stage. We are adding to this evidence with papers published since these reviews. This review has also included more mental health categories than other reviews to date. In addition, this is a scoping exercise focused solely on clinical utility of serious games. Other reviews have focused on a range of other matters, such as costs, speed and challenges in implementation and user motivation.^13–16^ This review looks to highlight the clinical and technological implications and challenges to move serious games from ‘theory to bedside’.

This review aims to evaluate the evidence available on the effectiveness and acceptability of serious games in improving the symptoms of mental health disorders by examining the extent, range and nature of research activity undertaken to date, to summarise and disseminate research findings, and comment on its current suitability to apply to clinical practice.

## Method

The Preferred Reporting Items for Systematic Reviews and Meta-Analyses (PRISMA) scoping review statement^17^ was used as a guideline for conducting this study.

### Search and study selection

The search string was a combination of serious games-related terms such as ‘serious games’ and ‘videogames’, and mental health-related terms such as ‘mental health’, ‘depression', ‘anxiety’ and ‘schizophrenia’ (Appendix 1). English was the primary language and studies published or translated into English were used. The search carried out on 18 March 2020 included studies published from January 2016 to April 2020. This publication date range was chosen because this review aimed to update a similar review that reviewed papers from 2015 and earlier.^13^

Duplicate items were removed from the records identified through the literature search. The remaining items were screened on basis of title, abstract and keywords. Items were included if the following inclusion criteria were met: the intervention used a digital game delivered on any technical platform, the intervention targeted mental health disorders as a primary diagnosis and the study conducted was a randomised controlled trial (RCT). Studies were excluded that involved predominantly later-onset cognitive disorders such as dementia, or DSM-V Axis II personality-linked disorders such as borderline personality disorders and autism spectrum disorders. The identified records were assessed for eligibility.

### Data extraction and synthesis

The data extraction was done by a single author, and when there was doubt, a second author was consulted. A data extraction sheet was developed on Microsoft Excel 2010 for Windows, and extracted variables such as target group, recruitment, treatment type, outcome measures, guidance during intervention, setting of intervention, study conditions, attrition, results, title of the game used in the study, serious game type and purpose of the game.

### Quality assessment

The quality of the included studies was assessed with the Grading of Recommendations, Assessment, Development and Evaluations (GRADE) criteria. When using GRADE, the evidence is made less certain by assessing: risk of bias, imprecision, inconsistency, indirectness and publication bias.^18^ Each study was rated as very low, low, moderate or high.

## Results

### Study selection

The EMBASE (*n* = 217), Medline (*n* = 145) and PsycINFO (*n* = 151) searches returned 513 items. After removal of duplicates, 395 records remained. Initial screening of title, abstract and keywords excluded a further 376 articles, leaving 19 items. After applying the exclusion criteria, 14 studies were included in the review. See Appendix 2 for a flow chart of the study inclusion. The second author was consulted to clarify the inclusion of two studies^19,20^ and the exclusion of one.

### Study characteristics

The study characteristics and participants of the included studies are presented in Table 1. The significant outcomes and qualitative results of the studies included are provided in Table 2. The 14 included studies were conducted in various locations ranging from Europe (Belgium,^19,21^ France,^22,23^ Ireland,^22^ The Netherlands,^19–21,24,25^ Portugal,^26^ Spain^27,28^) to Asia (Turkey^29^) and the USA.^30,31^ A total of 1167 participants were included (591 in intervention groups and 576 in control groups), with a total sample size ranging from 22 to 174 participants. Four studies targeted anxiety.^20,25,30,32^ Three studies focused on attention-deficit hyperactivity disorder (ADHD).^19,21,27^ Depression was targeted in two studies.^24,31^ Schizophrenia was the subject of two studies.^28,29^ Alcohol use disorder was targeted in two studies.^22,26^ Bipolar disorder was the focus of one study.^23^ Six studies were aimed at children,^19–21,25,27,30,32^ seven studies focused on adults^22–24,26,28,29^ and one study was aimed at older adults.^31^ Six studies compared serious games with treatment as usual (TAU) or no intervention ^19,21,26,28–30^; four studies compared them with a game;^22,24,27,32^ and four studies compared them with a therapy, such as CBT or a psychoeducational programme.^20,23,25,31^ The serious games were played in various settings: six studies were conducted at home,^19,21,24,27,29^ three at school,^20,25,32^ two at hospital,^22,29^ two at a clinic^26,28^ and two were unclear.^23,31^

**Table 1:** Characteristics of the randomised controlled trials that are included in the review

Reference, year, country	Target group	Game title	Game format	Recruitment	Primary outcome measures	Setting	Study conditions	*n* (% male)	Post-treatment and follow-up assessments	Study attrition (%)	GRADE
Anguera et al^31^, 2017, USA	Adults with late-life major depression	Project: EVO	Goal-oriented and problem-solving	Unclear, community-based patients	HRSD, PHQ-9, WHODAS-II, TOVA and delayed recognition task	Unclear, assumed home	1. Serious game 2. PST	1. 12 2. 10 (27%)	Post-treatment: 4 weeks; follow-up: 4 weeks	Post-treatment: 4.6%	Moderate
Bul et al^21^, 2016, The Netherlands and Belgium	Children with ADHD	Plan-It Commander	Goal-oriented and problem-solving	Mental healthcare clinics	Parent-reported time-management skills, planning/organising skills and cooperation skills	Home	1. Serious game 2. TAU	1. 88 2. 82 (81%)	Post-treatment: 10 weeks; follow-up: 10 weeks	Post-treatment: 10.6; follow-up: 18.2	Moderate
Bul et al^19^, 2018, The Netherlands and Belgium	Children with ADHD	Plan-It Commander	Goal-oriented and problem-solving	Mental healthcare clinics	Identify subgroups that were likely to get a high benefit from the treatment compared with the estimated treatment effect of the total group	Home	1. Serious game 2. TAU	1. 88 2. 82 (81%)	Post-treatment: 10 weeks; follow-up: 10 weeks	Post-treatment: 10.6; follow-up: 18.2	Moderate
Flaudias et al^22^, 2020, France	Adults with alcohol use disorder and attentional bias score >0	Cognitive bias modification game	Cognition training	University hospital	Hayling Test, AST, Classical Stroop, OCDS	Hospital	1. Serious game 2. Card-pairing game 3. Attentional bias score 0 ≥ playing serious game	1. 18 2. 15 (91%) 3. 8	Post-treatment:3 weeks	Unclear	Low
Gamito et al^26^, 2016, Portugal	Adults with alcohol dependence syndrome	Cognitive stimulation programme with several serious games	Cognition training	Private clinic	MMSE and FAB	Clinic	1. Serious game 2. Waiting list	1. 22 2. 23 (90%)	Post-treatment: 4 weeks	Post-treatment: 6.76	Very low
García-Baos et al^27^, 2019, Spain	Children with ADHD	RECOGNeyes using eye-tracking software	Cognition training	Unclear	Probability and severity of ADHD, hyperactivity index, impulsivity index, dyslexia index, performance parameters in two separate tests, usability and enjoyability	Home	1. Serious with eye-tracking 2. Serious game with mouse	*n* = 28 (64%)	Post-treatment: 3 weeks	Unclear	Low
Gülkesen et al^29^, 2017, Turkey	Adults with schizophrenia	ILFE	Cognition training	Unclear	Correct identification of facial expressions	Home and hospital	1. Serious game 2. Unclear, assumed TAU	1. 18 2. 14 (38%)	Post-treatment: 4 weeks	Unclear	Very low
Lado-Codesido et al^28^, 2019, Spain	Adults with schizophrenia	VOICES	Cognition training	Hospitals	RMV-SV	Clinic	1. Serious game 2. TAU	1. 27 2. 26 (52%)	Post-treatment: 4 weeks	Post-treatment: 5.7	Moderate
Maurin et al^23^, 2020, France	Euthymic adults with bipolar disorder	BIPOLIFE	Goal-oriented and problem-solving	Hospital	MARS	Unclear	1. Serious game and prior psychoeducational programme 2. Psychoeducational programme and TAU	1. 20 2. 21 (37%)	Post-treatment: 1 month; follow-up: 4 months	Post-treatment: 2.4; follow-up: 7.3	High
Sanchez et al^30^, 2017, USA	Children with social skills challenges	Adventures aboard the S.S. GRIN	Goal-oriented and problem-solving	Email, social media, schools	ALQ, social self-efficacy, social anxiety, social dissatisfaction, PECK and bullying perpetration	Home	1. Serious game 2. Waiting list	1. 33 2. 36 (60%)	Post-treatment: 9 weeks; follow-up: 1 week	Post-treatment: 26	Moderate
Schoneveld et al^32^, 2016, The Netherlands	Children with elevated anxiety	MindLight	Goal-oriented and problem-solving	Schools	SCAS, time spent playing video games, programme expectations and game evaluations	School	1. Serious game 2. Avatar game	1. 68 2. 66 (46%)	Post-treatment: 5 sessions; Follow-up: 3 months	Post-treatment: 8.8; follow-up: 15.4	Low
Schoneveld et al^25^, 2018, The Netherlands	Children with elevated anxiety	MindLight	Goal-oriented and problem-solving	Schools	SCAS, time spent playing video games, programme expectations and programme ratings	School	1. Serious game 2. Online CBT	1. 86 2. 88 (41%)	Post-treatment: 6 weeks; follow-up: 3 months, 6 months	Post-treatment: 17.2; follow-up: 3.4	High
Semkovska and Ahern^24^, 2017, Ireland	Currently remitted adults with a history of depression	RehaCom	Cognition training	Primary healthcare sites	Neurocognitive functioning	Home	1. Serious game 2. Online games	1. 11 2. 11 (18%)	Post-treatment: 5 weeks	Post-treatment: 4.5%	High
Wols et al^20^, 2018, The Netherlands	Children with elevated anxiety	MindLight	Goal-oriented and problem-solving	Schools	How SCAS scores related to in-game play behaviours and if changes in in-game play behaviours predicted changes in anxiety symptoms	School	1. Serious game 2. Online CBT	1. 86 2. 88 (41%)	Post-treatment: 6 weeks; follow-up: 3 months, 6 months	Post-treatment: 17.2; follow-up: 3.4	Moderate

GRADE, Grading of Recommendations, Assessment, Development and Evaluations; HRSD, Hamilton Rating Scale for Depression; PHQ-9, Patient Health Questionnaire; WHODAS-II, World Health Organization Disability Assessment Schedule II; TOVA, Test of Variables of Attention; PST, problem-solving therapy; ADHD, attention-deficit hyperactivity disorder; TAU, treatment as usual; AST, alcohol Stroop test; OCDS, Obsessive Compulsive Drinking Scale; MMSE, Mini-Mental Examination Test; FAB, Frontal Assessment Battery; ILFE, I am Learning Facial Expressions; RMV-SV, Reading the Mind in the Voice – Spanish Version; MARS, Medication Adherence Rating Scale; ALQ, Achieved Learning Questionnaire; PECK, Personal Experiences Checklist; SCAS, Spence Children’s Anxiety Scale; CBT, cognitive–behavioural therapy.

**Table 2:** Significant outcomes and qualitative results of the studies

Mental disorder	Number of studies	Outcomes favouring serious game	Outcomes favouring control
Anxiety	4	Sanchez et al^30^: Improvement by time compared with control, in social literacy (*P* = 0.000), social satisfaction (*P* = 0.039), social anxiety (*P* = 0.047) and bullying victimisation (*P* = 0.001). Schoneveld et al^32^: Improvements in anxiety symptoms by 3-month follow-up (*P* < 0.05). Schoneveld et al^25^: No significant differences between groups in anxiety symptoms. Non-inferiority for change in symptoms: serious game not worse than control for change in total reported anxiety symptoms at post-treatment, 3-month or 6-month follow-up; serious game not worse than control for personalised anxiety at 3-month follow-up, but significant differences at post-treatment and 6-month follow-up. Rated serious game and control equally appealing to themselves and appealing to others. No difference between groups for difficulty or anxiety inducing.^25^ Wols et al^20^: Engaged in-game play behaviours predicted significant improvements in anxiety symptoms at 3-month follow-up (*P* < 0.05). Avoidant/safety in-game play behaviours, significantly predicted higher anxiety symptoms at 3-month follow-up (*P* < 0.01).	Schoneveld et al^32^: Improvements in anxiety symptoms by 3-month follow-up (*P* < 0.05). Serious game rated significantly more anxiety-inducing than control (*P* < 0.05). Control game rated at post-treatment (*P* < 0.001) and follow-up (*P* < 0.05) as more appealing to themselves; at follow-up (*P* < 0.05) as more appealing to others; and at post-treatment (*P* < 0.05) as more likely to induce feelings of flow. Schoneveld et al^25^: CBT control rated significantly more relevant to daily life.
ADHD	3	Bul et al^21^: Improvements in parent-rated time-management skills (*P* = 0.004) in the serious game group. Secondary outcomes were parent-reported working memory (*P* = 0.02) and responsibility skills (*P* = 0.04) in the serious game group. Bul et al^19^: Significantly better planning and organising skills found in two subgroups: Girls (*n* = 26); and boys with low hyperactivity and higher conduct disorder score (*n* = 47). García-Baos et al^27^: Improvements from pre- to post-test and compared with the control for impulsivity index (*P* = 0.0067), number of fixations (*P* < 0.05), duration of fixations (*P* < 0.01) and reaction time (*P* < 0.0001). Children <12 years: 78% enjoyed the game; 66% found the difficulty appropriate; 44% found the challenge appropriate. Children ≥12 years: 50% did not enjoy the game; 40% had no specific opinion on enjoyability; 60% rated the game as not difficult; 60% rated it as not challenging.	Bul et al^21^: Higher drop-out rate in the intervention, those who dropped out had higher ADHD severity scores. Most common reasons for drop-out: child not motivated to play the game (*n* = 10) and difficulty level too high (*n* = 3). Adverse events reported in ten participants included pain in the fingers, irritability and headache.
Depression	2	Anguera et al^31^: Improvement for reported disability in both groups across time (*P* = 0.002). Improvements in working memory, with and without distraction (*P* < 0.05). Comparable improvements in serious game and control for depressive symptoms at post-treatment and 8-week follow-up (*P* < 0.05). 100% adherence to the intervention. Semkovska and Ahern^24^: Improvements compared with control in three targeted neurocognitive domains: divided attention (*P* = 0.016), verbal working memory (*P* = 0.003) and planning (*P* = 0.001). Improvements in non-targeted domains of long-term verbal memory (*P* < 0.001) and mental flexibility (*P* = 0.014).	Anguera et al^31^: Significant improvement for reported disability in both groups across time (*P* = 0.002). Semkovska and Ahern^24^: No difference in adherence between groups.
Schizophrenia	2	Gülkesen et al^29^: Improvement in identifying the correct facial expression compared with the control (*P* < 0.001). Lado-Codesido et al^28^: Improvements in RMV-SV score compared with the control (*P* < 0.001), and improvement from the pre-test to the post-test scores (*P* = 0.009); 80% of participants rated the serious game with ≥4 out of 5 points in the easy intelligibility and entertainment items.	Gülkesen et al^29^: Seven patients said the games were too easy.^29^ Lado-Codesido et al^28^: The serious game duration was scored 2.75 out of 5 points.^28^
Alcohol use disorder	2	Flaudias et al^22^: Improvement in capacity to inhibit appropriate automatic semantic response (*P* = 0.006), craving (*P* < 0.001) and attentional bias (*P* = 0.001).^18^ Gamito et al^26^: Improvement in frontal lobe functions (*P* = 0.001).	Flaudias et al^22^: Improvement in attentional control (*P* = 0.047) and craving (*P* = 0.003).
Bipolar disorder	1	Maurin et al^23^: Significantly higher absolute variation in medication adherence (*P* = 0.03) and in the secondary outcome, attitude toward psychiatric medications, (*P* = 0.002) after 1 month, which was no longer significant at 4 months; 82% in intervention group found content informative and thought that it would be relevant to others, 76% thought that information from the game would be useful. Patients reported that the actions of the avatar were limited and repetitive.^23^	Maurin et al^23^: One participant had a depressive episode during study.^23^

CBT, cognitive–behavioural therapy; ADHD, attention-deficit hyperactivity disorder; RMV-SV, Reading the Mind in the Voice – Spanish Version.

### GRADE rating

Table 3 shows the GRADE rating of each study. The GRADE criteria allocation was done by using the five factors of likely risk of bias, precision, consistency, directness and lack of publication bias. Specifically, tools for risk of bias and precision were investigated for. Presence of four or more factors gave a score of ‘high’, three factors gave a score of ‘moderate’, two factors gave a score of ‘low’ and one or no factors gave a score of ‘very low’. Three studies were rated as high.^23–25^ Six studies were rated moderate.^19–21,28,30,31^ Three studies were rated as low.^22,27,32^ Two studies were rated as very low.^26,27^

**Table 3:** GRADE rating criteria and individual study scores

Study	Overall GRADE score	Risk of bias	Imprecision	Inconsistency	Indirectness	Publication bias
Anguera et al^31^	Moderate	Moderate	High	High	High	High
Bul et al^21^	Moderate	Moderate	High	High	High	High
Bul et al^19^	Moderate	Moderate	High	High	High	High
Flaudias et al^22^	Low	Low	High	High	High	High
Gamito et al^26^	Very low	Low	High	High	Moderate	High
García-Baos et al^27^	Low	Low	High	High	High	High
Gülkesen et al^29^	Very low	Low	High	High	Moderate	High
Lado-Codesido^28^	Moderate	Moderate	High	High	High	High
Maurin et al^23^	High	High	High	High	High	High
Sanchez et al^30^	Moderate	Moderate	High	High	High	High
Schoneveld et al^32^	Low	Moderate	High	High	Moderate	High
Schoneveld et al^25^	High	High	High	High	High	High
Semkovska and Ahern^24^	High	High	High	High	High	High
Wols et al^20^	Moderate	Moderate	High	High	Moderate	High

GRADE, Grading of Recommendations, Assessment, Development and Evaluations.

The results have been divided into subheadings of the mental disorders targeted in the studies.

### Anxiety

Four studies targeted symptoms of anxiety or social skills in children. Three used the same serious game MindLight.^20,25,32^ Two of these studies^20,25^ were the same, but one had different outcomes measured at a later time.^20^

MindLight is a three-dimensional, third-person neurofeedback video focused on a boy saving his grandmother from evil forces at her scary mansion (see video of game trailer: https://www.youtube.com/watch?v=buNaErarLts). In all three studies the game was played at school, after hours. MindLight was defined as a goal-oriented and problem-solving type of serious game.

In one of the MindLight studies, the games were played in an unspecified number of groups of 7–19 children (aged 8–13 years, 46% male, 68 in the intervention group, 66 in the control group), who were all supervised by two research assistants. The rationale for the age group of children targeted was not provided. The sessions were 1 h long, two times a week, and they had five sessions in total. The rationale for the dose was not provided. The control group played Max and the Magic Marker, a puzzle platform video game involving a small vulnerable boy overcoming in-game challenges (see video of game trailer: https://www.youtube.com/watch?v=1MPGb7akIpc). An active control was used to ensure that attention, motivation, behavioural activity and expectations do not account for improvements in the intervention group. Both games are valid for the population chosen, as they are designed for children, and MindLight specifically targets children with anxiety symptoms. The participants and assistants running the sessions were not blinded. The randomisation of participants was stratified by gender and school grade, and was performed by an independent researcher using the SPSS random number generator. There was no presence of a conflicts of interest section in the paper, so it is unclear whether there were any or not. The outcomes were measured using questionnaires on anxiety symptoms (parent and child version of Spence Children’s Anxiety Scale; SCAS), average time spent playing video games, game expectations and game evaluations (appeal to self, appeal to others, relevance, flow and difficulty).^32^

In this first study when compared with the avatar game, both MindLight and the control group showed improvements in anxiety symptoms at the 3-month follow-up (*P* < 0.05). The control group also was rated as more appealing to themselves at post-treatment (*P* < 0.001) and follow-up (*P* < 0.05), more appealing to others at follow-up (*P* < 0.05) and more likely to induce feeling of flow at post-treatment (*P* < 0.05).^32^

The other two studies played MindLight in an unspecified number of groups of five to ten children (aged 7–12 years, 40.8% male, 86 in intervention groups, 88 in control groups) supervised by Master's students for 1 h a week, for 6 weeks. The rationale for the age group of children targeted was not provided. The control group did CBT as Coping Cat (an effective CBT programme for anxious children) for eight sessions after school, in groups of four to seven children led by two psychologists.^20,25^ The rationale for the dose was not provided. The participants and assistants running the sessions were not blinded. The randomisation of participants was stratified by gender and school grade, and was performed by an independent researcher using the SPSS random number generator. One of the authors of these two studies is the founder of the PlayNice Institute, the executive producer of the MindLight game.^20,25^

The outcomes from the second study using MindLight were measured with questionnaires on total anxiety (parent and child SCAS), personalised anxiety, total hours per week playing video games, programme expectations and programme ratings (appeal to self, appeal to others, relevance, anxiety inducing and difficulty).^25^

Compared with online CBT, there was child- and parent-reported decreases in anxiety levels in children for up to 6 months. MindLight was also rated as non-inferior to the CBT control, suggesting that it is a sufficient alternative to traditional CBT. However, the CBT control was rated significantly more relevant to daily life.^25^ The scale of difficulty of MindLight as a game was rated well by children; it was at the middle of the scale, suggesting that it has a good balance of learning and challenge.^25^

These two studies^20,25^ were the same, but one had different outcomes measured at a later time.^20^ The outcomes from the third study using MindLight included how pre-test anxiety scores were related to in-game play behaviours during the first play session, and whether changes in in-game play behaviours from the first to last play session predicted changes in anxiety symptoms at the 3-month follow-up. The coding was done by research assistants that were blinded to the hypotheses.^20^

There were significant improvements in anxiety symptoms at the 3-month follow-up (*P* < 0.05) for children with engaged in-game play behaviours. There were significantly predicted higher anxiety symptoms at the 3-month follow-up (*P* < 0.01) for children with avoidant/safety in-game play behaviours.^20^

The fourth study used the serious game ‘Adventures aboard the S.S.GRIN’ in children with social skills challenges (aged 7–11 years, 60% male, 33 in the intervention group, 36 in the control group) (see video of game trailer: https://www.youtube.com/watch?v=fHEJs8gr7mk). The rationale for the age group of children targeted was not provided. The game was developed from skills taught in an evidence-based, in-person social skills programme,^33^ and involves using a personalised avatar to interact with non-playable characters on the sailing ship S.S. GRIN. There was in-game feedback, hints and prompts based on performance. Weekly episodes were released for a total of 9 weeks, and players had 1 week to complete each episode. The rationale for the dose was not provided. The game was specifically designed for children with social skills challenges. Adventures aboard the S.S.GRIN was defined as a goal-oriented and problem-solving type of serious game. The relevant subscales of the Behaviour Assessment System for Children, Second Edition (BASC-2) norm-referenced screening system for measuring behavioural and emotional strengths and weaknesses was used to identify children struggling with socioemotional skills. The rationale for the dose was not provided. The control group was placed on a waiting list, but it was not made clear why this decision was made.^30^

The participants and the assistants running the sessions were not blinded. The randomisation of participants was stratified by age, gender, race, ethnicity and BASC-2 subscale scores. The authors were employees of the 3C Institute, which may benefit financially from game sales. The outcomes were measured using questionnaires on social literacy (Achieved Learning Questionnaire); social self-efficacy (Self-Efficacy and Outcome Expectancy Measure); social anxiety (Social Anxiety Scale for Children – Revised); social satisfaction (Loneliness and Social Dissatisfaction Scale); bullying victimisation (Personal Experiences Checklist); and bullying perpetration (bullying others subscale of the California Bullying Victimisation Scale).^30^

This game appeared to be effective as, compared with the control group, there were improvements in social literacy (*P* = 0.000), social satisfaction (*P* = 0.039), social anxiety (*P* = 0.047) and bullying victimisation (*P* = 0.001). However, the control group was a waiting list and an active control was not used, meaning that the results could just reflect the effect of gaming.^30^

### ADHD

Three studies targeted symptoms related to ADHD in children.^19,21,27^

Two papers used the same serious game ‘Plan-It Commander’^19,21^ (see video of game trailer: https://www.youtube.com/watch?v=8FoSDUJS8eQ&ab_channel=%26ranj). These two papers performed the same study, but the later paper separately analysed a subgroup from the original data at a later time.^19^ Plan-It Commander is an online adventure game designed for children with ADHD, and was developed by healthcare professionals, researchers and game experts to improve domains of daily life functioning, with a primary focus on time management, planning/organising and cooperation skills. The game had a closed social community of players, and had a competitive element where players could gain badges and gifts. The study focused on children aged 8–12 years, 81% were male and there were 88 in the intervention group and 82 in the control group. The rationale for the age group of children targeted was not provided. The game was played at home and players were instructed to play for a maximum of 65 min, approximately three times a week for 10 weeks. The rationale for the dose was not provided. The game was specifically designed for children with ADHD. Plan-It Commander was defined as a goal-oriented and problem-solving type of serious game. The control group received TAU; it was not made clear why this decision was made.^19,21^

The participants were not blinded, and it was not possible to blind researchers and teachers. The randomisation of participants was done on a 1:1 ratio by a prespecified, computer-generated randomisation list, and the allocation was stratified by study site and gender. The authors reported that there were no conflicts of interest. The primary outcomes for the first study were online questionnaires done by parents on reported time-management skills, planning/organising skills (the subscale Plan/Organize of the Behavior Rating Inventory of Executive Function, parent version) and cooperation skills (the subscale Cooperation of the Social Skills Rating System, parent version).^21^

The outcome of the second study was to use a virtual twins analysis to identify subgroups that were likely to get a high benefit from the treatment compared with the estimated treatment effect of the total group.^19^

Plan-It Commander showed improvements in multiple domains: parent-rated time-management skills (*P* = 0.004), parent-reported working memory (*P* = 0.02), and responsibility skills (*P* = 0.04), suggesting that it is an effective intervention. However, there was a higher drop-out rate in the intervention and those who dropped out had higher ADHD severity scores. This suggests that this serious game may not be suitable for children with more severe ADHD.^21^

Furthermore, in the follow-up study, it was found that there were significant improvements in planning and organising skills in two subgroups: girls, and boys with low hyperactivity and higher conduct disorder scores.^19^

The other study used the serious game RECOGNeyes in children (age 8–15 years, 64% male, 28 total participants). The rationale for the age group of children targeted was not provided. This game involved the player using eye-tracking software to catch snowflakes while avoiding fire in six subgames. The game was installed on provided laptops and played at home. The game was played for 30 min, three times a week for 3 weeks. The rationale for the dose was not provided. The game is specifically designed for training attention in people with ADHD. RECOGNeyes was defined as a cognition training type of serious game. The control group played the same game but using a mouse instead of eye-tracking software. The reason for this was not fully explained.

There was no description of blinding within the study. The description of the randomisation was unsatisfactory as the paper only stated that the groups were balanced for comorbidities and gave no more details. Two of the authors were affiliated with Braingaze SL, the eye-tracking software company, and one of these authors received money from Braingaze SL to carry out the study. The outcomes were assessed using a Frog task to assess the probability of ADHD, severity of ADHD, hyperactivity index, impulsivity index and performance parameters (number of errors, reaction time, and number and duration of gaze fixations). A word recognition task assessed the dyslexia index and performance parameters (number of errors, reaction time, and number and duration of gaze fixations). A questionnaire with a three-point scale was used to assess usability and enjoyability. It was not stated if this was a validated questionnaire.^27^

The results from the serious game RECOGNeyes that used eye-tracking software appeared to show that it was effective; however, the study was given a ‘low’ GRADE score and there were many elements of the study that were not sufficiently described. This serious game could still have the opportunity to be shown to be effective in future studies with a more robust study design.^27^

### Depression

Two studies targeted depression.^24,31^

One study used the game Project: EVO on a mobile or iPad, which involved guiding a character thorough an immersive environment and responding to targets. Project: EVO was defined as a goal-oriented and problem-solving type of serious game. The game was played by adults (mean age 68 years, s.d. 6.3, 27% male, 12 in the intervention group, ten in the control group) and they were instructed to play for 20 min, five times a week for 1 month. The 4-week duration of the intervention was chosen because it was based upon the previous duration used by the authors’ previous study that the serious game in this study was derived from.^34^ The duration of the trial was based on a previous similar study. The game was not designed for this population and was originally designed for children. The control group received problem-solving therapy, an intervention that helps patients with goal-directed behaviour, for 8 weeks; the rationale for this choice was not clear. There was no description of blinding within the study. The randomisation was done by a random number generator. The authors reported that there were no conflicts of interest.

The outcomes were collected by research assistants on depression symptoms (Hamilton Rating Scale for Depression and Patient Health Questionnaire-9), physical disability (Charlson Comorbidity Index), sustained attention (test of variables of attention), working memory (delayed recognition task), a basic response time task and a trait adjective task to assess negativity bias.^31^

The serious game showed improvements in working memory, with and without distraction (*P* < 0.05), but comparable improvements to the control in reported disability (*P* = 0.002) and depressive symptoms post-treatment and at the 8-week follow-up (*P* < 0.05). There was, however, full adherence to the intervention.^31^

The second study use a game called RehaCom, a computerised neurocognitive remediation therapy in adults (aged 31–65 years, 18% male, 11 in the intervention group, 11 in the control group). The game had six procedures, all with multiple levels of difficulty: divided attention (1 and 2), verbal memory, figural memory, shopping and plan a day. The players downloaded the game on their personal computers and played at home. The control group played games that required selective attention, strategy and remembering cues. Both groups had to play for 1 h, four times a week for 5 weeks, and were sent random reminders to complete their sessions. This study followed the same research protocol as a previous study of one of the authors.^35^ The reason for the choice of control was not fully explained. The game is designed for neurocognitive remediation therapy. RehaCom was defined as a cognition training type of serious game.

The participants were not blinded but the researchers were blinded. The group allocation was stratified by gender and generated by a pre-programmed command with the statistical package R. The authors reported that there were no conflicts of interest. The outcome for this study was neurocognitive functioning, which was assessed by a standardised battery of validated neuropsychological tests (the Digit Symbol Substitution to assess psychomotor processing speed; the d2 Test of Attention for divided attention; the Digit Span Forward for auditory attention; the Digit Span Backward for verbal working memory; the Logical Memory I and II for verbal learning and retention, respectively; the Rey–Osterrieth Complex Figures for visual learning (immediate recall) and retention (delayed recall); and the following Delis–Kaplan Executive Function System subtests for the assessment of the stated executive functions: Verbal Fluency (three consecutive categories) for self-regulation under external constraints, Fluency Switching for mental flexibility, Towers for planning and 20-Questions for abstract thinking.^24^

It is important to note that one study solely focused on neurocognitive functioning,^24^ which is just one of the several deficits seen in patients with depression. The serious game in this study, RehaCom, showed multiple improvements in the intervention group compared with the control group: divided attention (*P* = 0.016), verbal working memory(*P* = 0.003), planning (*P* = 0.001), long-term verbal memory (*P* < 0.001) and mental flexibility (*P* = 0.014). Allowing this serious game to be played at home, when it is most convenient and potentially comfortable for the patient, could lead to improved adherence in these patients.^24^

### Schizophrenia

Two studies targeted symptoms of schizophrenia.^28,29^

One study used a game called I am Learning Facial Expressions (ILFE) in adults (mean age 37.3 years, s.d. 9.2, 38% male, 18 in the intervention group, 14 in the control group). This included eight serious games with a range of levels. The player was shown different images of people’s faces and had to correctly identify the shown emotion. Those without a computer at home played using a hospital computer, and if patients forgot to play then they were reminded. They were instructed to play for 1 h, at least two times a week for 1 month. The rationale for this dose was not provided. The control group is assumed to be TAU, but was not sufficiently described. The game is designed with a consideration for some of the common characteristics of schizophrenia. ILFE was defined as a cognition training type of serious game. The reason for the chosen dosage was not made clear. There was no mention of blinding in the study. The group allocation was randomised. The authors reported that there were no conflicts of interest. The primary outcome was correct identification of facial expressions.^29^

The serious game ILFE appeared to be effective at improving the correct identification of facial expression compared with the control (*P* < 0.001). However, the reliability of this study is questionable, as it was given a GRADE score of ‘very low’.^29^

The second study used the serious games VOICES in adults (mean age 40.9 years, s.d. 12.1, 52% male, 27 in the intervention group, 26 in the control group). The game involved the patient being played a phrase and selecting the emotion conveyed. The patients attended a centre and played in a quiet room with trained personnel there to show them how to use the game. They played for approximately 30 min, two times a week for 1 month, and each session increased in difficulty. The rationale for this dose was not provided. The control received TAU; the reason for this was unclear. The game is designed to train emotions. VOICES was defined as a cognition training type of serious game. The reason for the chosen dosage was not made clear. The participants were not blinded but the researchers were blinded. The group allocation was done by a computer-generated randomisation list. There was no presence of a conflicts of interest section in the paper, so it is unclear whether there were any or not.

The primary outcome was emotion recognition using the Reading the Mind in the Voice – Spanish Version test.^28^

The other study, which used the serious game VOICES, appeared to be effective as there was a significant improvement from the pre-test to the post-test scores (*P* = 0.009), and also a significant improvement compared with the control (*P* < 0.001).^28^

For adults with schizophrenia, it is important to note that one study solely focused on the correct identification of facial expressions^29^ and the other study focused on emotion recognition from spoken words.^28^ These two outcomes measured will reflect only two of the several deficits seen in patients with schizophrenia.

### Alcohol dependence

Two studies targeted symptoms of alcohol dependence.^22,26^

One study used a game based on a cognitive–behavioural modification programme on a tablet device in adults (intervention group mean age 48.5 years, s.d. 12.63; control group mean age 43.73 years, s.d. 7.86; 91% male; 18 in the intervention group; 15 in the control group). The game involved a series of pictures with four images shown at once, of which three were alcohol-related images and one was not. The patients needed to choose the unrelated picture as quickly as possible. The game was played in groups of four to five for 30 min, two times a week for 3 weeks. The rationale for the dose was not provided except for one reference to a previous study.^36^ This was compared with a control group that used a card-pairing memory game, which was used to avoid a potential effect of ‘gaming’ in the intervention. The game is designed for attentional bias training in patients with alcohol use disorder, and it was defined as a cognition training type of serious game. The reason for the chosen dosage was not made clear. There was no mention of blinding in the study. The groups were randomly allocated. The authors reported that there were no conflicts of interest. In this study, the outcomes were capacity to inhibit and appropriate automatic semantic response (Hayling Sentence Completion Test), attentional bias toward alcohol (alcohol Stroop test), attention control (classical Stroop test), severity of alcohol usage (Alcohol Use Disorders Identification Test) and craving (Obsessive Compulsive Drinking Scale).^22^ There were improvements in the intervention group in capacity to inhibit appropriate automatic semantic response (*P* = 0.006), craving (*P* < 0.001) and attentional bias (*P* = 0.001), but there were also improvements in the control group in attentional control (*P* = 0.047) and craving (*P* = 0.003). The GRADE score for this study was ‘low’, which makes the reliability of the results questionable.^22^

The second study used several serious games designed as a cognitive stimulation programme in adults (mean age 45.45 years, s.d. 10.31, 90% male, 22 in the intervention group, 23 in the control group). The difficulty increasing with each level and the games were designed to stimulate attention, memory, decision-making, language, processing speed, strategic planning, perception and spatial vision. Some of the games were developed by the team and some were available commercially, but were set up as a cognitive stimulation programme. The game was defined as a cognition training type of serious game. The games were played for 45–50 min, three times per week for 1 month, for ten sessions in total. The rationale for the dose was not provided. The control was placed on a waiting list and received TAU. The reason for the choice of control was not fully explained. There was no mention of blinding in the study. The groups were randomly selected. There was no presence of a conflicts of interest section in the paper, so it is unclear whether there were any or not. The primary outcomes for this study were general cognitive ability (Mini-Mental State Examination) and frontal lobe functions (Frontal Assessment Battery).^26^ The study showed improvements in frontal lobe functions (*P* = 0.001), but again the GRADE score for this study was ‘very low’, which makes this significant result very unreliable.^26^

### Bipolar disorder

One study targeted symptoms of bipolar disorder by using the game BIPOLIFE in adults (intervention group mean age 46.58 years, s.d. 14.01; control group mean age 41.44 years, s.d. 7.62; 37% male; 20 in the intervention group; 21 in the control group). This game is centred on an avatar with bipolar disorder in everyday situations, with the aim being for the player to regulate the avatar's mood. The participants were told to play as often and for as long they wanted to during a 1-month period, and there were in-game messages to prompt the player. Before the game, the patients received a 12-week psychoeducational programme. The control group received TAU and a 12-week psychoeducational programme. The rationale for the dose was not provided. The reason for the choice of control was not fully explained. The game is designed for patients with bipolar disorder. BIPOLIFE was defined as a goal-oriented and problem-solving type of serious game.

The participants were not blinded, but the researchers were blinded. The allocation was done randomly and assigned with a 1:1 ratio, and the randomisation sequence was centralised and computed in blocks of four for each level of stratification by the study statistician, in an order unknown by the investigators. The study was funded by AstraZeneca, who were involved in the BIPOLIFE game development. The primary outcome of this study was medication adherence rate (Mediation Adherence Rating Scale), which was recorded by trained psychologists or psychiatrists.^23^

In the intervention there was significantly higher absolute variation in medication adherence (*P* = 0.03) and improvements in attitude toward psychiatric medications (*P* = 0.002) after 1 month, but this was no longer significant at the 4-month follow-up. These results should be reliable as the study was given a GRADE score of ‘high’. Encouraging positive thoughts and behaviours around BIPOLIFE as a treatment could lead to improved medication adherence in patients.^23^

## Discussion

The aim of this scoping review was to give an overview of serious games for mental health symptoms that were evaluated with RCTs. There were 11 different games in the review, focusing on anxiety, ADHD, depression, schizophrenia, alcohol dependence and bipolar disorder. The games were categorised as either goal-oriented, problem-solving or cognitive/brain training. Five games (Project: EVO, Plan-It Commander, BIPOLIFE, Adventures Aboard the S.S. GRIN and MindLight) were categorised as both goal-oriented and problem-solving games. Six games (cognitive bias modification game, cognitive stimulation programme with several serious games, RECOGNeyes, ILFE, VOICES and RehaCom) were categorised as cognition training games. More exploration and further similar studies are required to determine which genre is optimal for specific mental disorder symptoms.

There was a more even balance of games available to play on a PC or a tablet compared with a previous review on serious games in mental health.^37^ With an increase in sales of tablets and smartphones, more people spend time using these devices and playing games on them. Developing serious games to be downloaded onto this platform is important to increase adherence in the desired population. There are many mental health apps available, and incorporating serious games can provide many opportunities.^38^ This research is at an early stage, with relatively few RCTs in several different mental health conditions. However, early results from the RCTs give grounds for optimism that serious games may offer a significant new treatment for mental illnesses. As the research develops, key aspects need to be well-defined, including the gaming intervention, control group intervention, illness severity and social acceptability of the intervention (e.g. if it should be delivered at home or in the hospital).

The studies performed with children focused solely on ADHD or anxiety symptoms.^19–21,25,27,30,32^ Only one of the studies in children used a game targeted for cognitive training.^27^ For the control groups there was a mix of no added intervention,^19,21,26,28–30^ alternative games^22,24,27,32^ and an added therapy.^20,23,25,31^ No serious games primarily targeting obsessive–compulsive disorder or post-traumatic stress disorder were found in this review. An explanation for this could be that game elements are not necessary to add to virtual reality exposure therapy, which already exists to combat symptoms of these disorders.

### Strengths and limitations

The strength of this study is that it provides an up-to-date insight into the potential effectiveness of serious games on the symptoms of mental disorders based on RCTs – the gold standard of research – and expand on already provided insights in previous reviews. A limitation of this review is that only one person, instead of two, reviewed and performed the data extraction on the papers. Furthermore, five studies had a GRADE rating of low or very low, which indicates that the methodological quality and reporting of the studies could be improved. The studies were published in multiple non-English-speaking countries, meaning that some of the extracted data for this review was from translated papers. This is a minor limitation as the translation are expected to be reliable and accurate.

This review focuses solely on serious games and not on the related field of gamification. At present, there is a conceptual ambiguity in the use of the two, but there is a defined need to report these interventions separately.^39^ Although both are considered streams of CBT, gamification differs from serious games in domains of game mechanics, competition, reward system and primary purpose.^39–41^ Whereas serious games has focused learning goals and objectives, gamification is about enhancing user engagement for more broad-based e-learning.^39–42^

It is worth noting that in the past decade there have been several other high-quality reviews.^13–16^ These reviews, although largely on the same subject, had different search strategies and design. One comprehensive review offered a large and relatively similar pool of studies as the current study.^13^ Our review is narrower as it looks only at serious games and not gamification. This review looked to understand if there is further evidence to update this topic. It is worth recognising that serious games is a fast-moving area. The focus of the review was on using serious games in clinical practice. A comparison of our review with other similar previous reviews is offered in Table 4.

**Table 4:** Comparison of current review with other previous reviews

Review	Methodology	Scope of review	Clinical area	Intervention	Number of studies	Conclusion
Fleming et al, 2014^16^	Systematic review	Systematic review of serious games for the treatment and prevention of depression	Depression	Serious games	9	Interventions promising but evidence limited
Li et al, 2014^15^	Systematic review and meta-analysis	Systematic review and meta-analysis of game-based digital interventions on the effectiveness and effect size in depression	Depression	Game-based digital interventions	19	Findings support effectiveness
Li et al, 2016^14^	Systematic review and meta-analysis	Systematic review and meta-analysis of exergames on the effectiveness and effect size in depression	Depression	Exergames	9	Small but significant positive effect of exergames
Fleming et al, 2017^13^	Scoping review	Scoping review exploring the potential and opportunities of serious games and gamification. Review of systemic reviews identifying systematic reviews of depression	Mental health	Serious games and gamification	Not applicable	Applied games have considerable potential to help mental health, direct comparisons between game-based interventions and non-game based interventions lacking
This review	Scoping review	Clinical effect of serious games as a treatment	Mental illnesses including depression, anxiety, attention-deficit hyperactivity disorder, alcohol dependence bipolar disorder, schizophrenia	Serious games	14	See Discussion

### Implications for clinical practice

Serious games are a potentially cost-effective intervention for patients, and early RCTs are promising. In healthcare services under extreme pressure because demand for treatment exceeds the supply, any cost-effective treatment that is acceptable to patients needs to be explored. Clinicians tend to be more comfortable with treatments they are trained in, whether that is talking therapies, social interventions or biological treatments, but there needs to be an active debate on how serious games fit into clinical management plans once an established research base justifies their use. An independent authority without commercial interests is needed to recommend which games are effective, as some studies are financed by gaming companies or pharmaceutical companies with clear financial interest in their success.

There are several points to be taken away from this review and put into use by clinicians. For example, regarding anxiety in children, a child may benefit more from a game if they are motivated to play, as they believe they will feel better afterward.^32^ Many children play games to reduce stress and build feelings of independence, and these are more pronounced in children with elevated mental health symptoms.^43^

### Implications for research

There is space for improvement in future studies on serious games in mental health disorders. First, performing new studies and repeating others in larger populations will allow them to have higher statistical power. Larger groups may also allow for more balanced groups on gender, which could lead to study differences.^22^ Furthermore, there should be extended follow-ups to provide a more reliable evaluation of benefits that develop or are maintained over time. Regarding the intervention itself, there are multiple adaptations that should be made. First, more investigation should be done for the optimal intervention dose, as it is unclear what the ideal intervention dose is for a particular population and particular disorder. This could confirm alterations in behaviour and symptoms; in one study of patients with schizophrenia, the intervention was delivered for a total of 4 h, which the patients rated as brief.^28^ It has been reported that in schizophrenia the optimum intervention time is 20 h to observe a significant difference in auditory processing speed.^44^

Some of the serious games in the studies were not designed for the disorder being treated, whereas in some studies, the game specifically targeted symptoms of the disorder. Targeting specific age groups could be important. In a study on children with ADHD, children under 12 years of age enjoyed the games the most, whereas children over 12 years of age did not rate the game highly. This suggests that this form of game was not suitable for the older children, and something more stimulating would need to be used.^27^ Also, for age groups, the adherence of older age groups should be compared with those of younger age groups to see whether the real-life application of the serious game would be useful. The odds of using the internet decreases with age, so some serious games may be better suited to a younger population.^45^ Furthermore, according to the results of one study, people with schizophrenia enjoy playing more complex games, and this should be explored by using games of varying complexity in this disorder.^29^ Another alteration would be to perform more trials where the serious game is played at home instead of at a centre or hospital, as this is likely to be the place where an accessible intervention of this form is most likely to be utilised and would reflect real-world relevance. This would also reflect whether spontaneous motivation to play improves outcomes.

It is important to identify which patients will benefit the most from this intervention. In one study of children with ADHD, the patients that dropped out had higher ADHD severity scores, suggesting that a serious game intervention may not be appropriate for them.^21^ A further example of this was a study of bipolar disorder, but it only included patients experiencing euthymia.^23^

The serious games reviewed in this paper generally have been tested against a TAU group. In such complex interventions, the TAU is vulnerable to significant placebo effects. Thus, to gain the confidence to use these interventions in clinical settings, more robust research testing needs to be done. In particular, to measure impact, the effect of the placebo needs to be carefully unpicked.^46^ Placebos are context-sensitive and can be a crucial issue to such interventions. Patient and clinician expectations increase the chance of wanting such interventions working. This can be further enhanced by the media and society. The placebo effect is particularly dependent on the participant–clinician link, along with the perceptions of the individual participant. It is worth considering that a placebo effect can occur when a participant experiences improvements in their condition despite receiving a placebo intervention. Similarly, an active comparison group (such as TAU) might not be the cause of a placebo effect. In such situations, other biases that might arise from using TAU can be explored. Conscious understanding and knowledge would be needed to mitigate these. An option would be to compare the serious game with the best evidence-based intervention for the targeted disorder, and run the trial as a non-inferiority study.

A further improvement would be to introduce more accurate measurements for the outcomes. Many of the studies in this review used questionnaires and self-reported measures, which are sometimes not truly representative. An idea to improve this could be when measuring medication adherence, instead of solely relying on patient-reported measures, there could also be plasma-level measurements or pill counts.

Another area that requires further inquiry is the role of serious games in various cognitive and personality-linked mental disorders, specifically later-onset cognitive disorders and DSM-V Axis II personality-linked disorders. Given the potential benefits, this is an area of further research.

### Implications for technology

For research studies to advance, the serious games themselves need to be improved. First, it is important to design or convert more serious games for use on tablets or smartphones rather than computers, as these are used more frequently and may increase the usage over time.^37^ There is a need to invest in research to measure the effectiveness by an industry that is not biased by its interests. Partnerships between leaders in health departments and the gaming industry may lead to increased development of serious games for mental health.

A further improvement would be to include the target audience in the game design process. This will make the serious games more relevant, appealing and engaging. Also, the commercial industry should be engaged in the game design alongside scientists, to make the serious game visually appealing, entertaining and scientifically validated for mental health. This could include making more games that connect to the internet and allow interaction and competition with other players. There is a massive potential for this to be a new, emerging treatment modality that is more acceptable, especially for younger people.

Moreover, the coding of the serious game can be improved to distinguish between play patterns of patients. This can be recorded and analysed as outcomes reflecting the behaviour of the player, and give a prognostic value. The code could also be used to modify the game scenario to best suit the players’ tendencies. An application of this could be that a child with anxiety may exhibit withdrawn play behaviours and the game could be coded to expose the player to elements that will benefit that individual the most.^20^

Findings from serious games might also be applicable among commercial games, and require further studies of commercial games effects on players’ mental health. Recently, there are indications of a modest positive co-relation between players’ perceptions, well-being and in-game behaviours becoming evident.^47,48^ This requires further inquiry.

In conclusion, we are at the very early stages of the evaluation of serious games as a treatment in the mental health setting. Although a promising area, the evidence available on the effectiveness and acceptability of serious games does not support immediate adoption into routine clinical practice. As studies develop, it is crucial to be clear on the interventions used (i.e. type of game, setting, dosage and study conditions), the diagnoses and the outcomes, as generalisation for health guidelines becomes difficult when these variables are made to be very specific. Although currently there is little evidence on this topic, findings point to serious games being effective as an intervention in reducing mental health problems. A meta-analysis is recommended to follow up on this review to identify the effectiveness of specific games to individual disorders, as well as further RCTs. Game development and design remains important, as well as making games available on a tablet or smartphone device. More investigation is needed into serious games that can connect with other players with an internet connection. Partnerships between the gaming industry and health research need to be established, with clear declarations of interest.

## Data Availability

The data that support the findings of this study are available from the corresponding author, R.S., upon reasonable request.

## Supplementary material

For supplementary material accompanying this paper visit http://doi.org/10.1192/bjo.2022.4. click here to view supplementary material

## Appendix 1 Search terms

PsycINFO Healthcare Databases Advanced Search

**Table:**

	Search term
1	(serious game*).ti,ab
2	(video game*).ti,ab
3	‘COMPUTER GAMES’/ OR ‘DIGITAL GAME-BASED LEARNING’/ OR ‘DIGITAL GAMING’/ OR ‘SIMULATION GAMES’/ OR ‘ROLE PLAYING GAMES’/
4	(2 OR 3)
5	(therap*).ti,ab
6	(4 AND 5)
7	(1 OR 6)
8	(mental health).ti,ab
9	exp ‘MENTAL HEALTH’/
10	(mental illness).ti,ab
11	exp ‘MENTAL DISORDERS’/
12	(depression).ti,ab
13	‘DEPRESSION (EMOTION)’/
14	(anxiety).ti,ab
15	exp ‘ANXIETY DISORDERS’/
16	(problem drinking).ti,ab
17	‘ALCOHOL USE DISORDER’/ OR exp ‘ALCOHOL ABUSE’/
18	(schizophrenia).ti,ab
19	SCHIZOPHRENIA/
20	(‘obsessive compulsive disorder’ OR ‘OCD’).ti,ab
21	‘OBSESSIVE COMPULSIVE DISORDER’/
22	(‘post-traumatic stress disorder’ OR ‘PTSD’).ti,ab
23	‘POSTTRAUMATIC STRESS DISORDER’/
24	(‘attention-deficit hyperactivity disorder’ OR ‘ADHD’).ti,ab
25	‘ATTENTION DEFICIT DISORDER WITH HYPERACTIVITY’/
26	8 OR 9 OR 10 OR 11 OR 12 OR 13 OR 14 OR 15 OR 16 OR 17 OR 18 OR 19 OR 20 OR 21 OR 22 OR 23 OR 24 OR 25
27	7 AND 26
28	27 [DT 2016-2020]

EMBASE Healthcare Databases Advanced Search

**Table:**

	Search term
1	(serious game*).ti,ab
2	(videogame*).ti,ab
3	‘VIDEO GAME’/ OR ‘VIDEO GAMES’
4	(2 OR 3)
5	(therap*).ti,ab
6	(4 AND 5)
7	(1 OR 6)
8	(mental health).ti,ab
9	‘MENTAL HEALTH’/
10	(mental illness).ti,ab
11	‘MENTAL DISEASE’/
12	(depression).ti,ab
13	DEPRESSION/
14	(anxiety).ti,ab
15	‘ANXIETY DISORDER’/
16	(problem drinking).ti,ab
17	ALCOHOLISM/
18	(schizophrenia).ti,ab
19	SCHIZOPHRENIA/
20	(‘obsessive compulsive disorder’ OR ‘OCD’).ti,ab
21	‘OBSESSIVE COMPULSIVE DISORDER’
22	(‘post-traumatic stress disorder’ OR ‘PTSD’).ti,ab
23	‘POSTTRAUMATIC STRESS DISORDER’/
24	(‘attention-deficit hyperactivity disorder’ OR ‘ADHD’).ti,ab
25	‘ATTENTION DEFICIT DISORDER WITH HYPERACTIVITY’/ OR ‘ATTENTION DEFICIT HYPERACTIVITY DISORDER’/
26	8 OR 9 OR 10 OR 11 OR 12 OR 13 OR 14 OR 15 OR 16 OR 17 OR 18 OR 19 OR 20 OR 21 OR 22 OR 23 OR 24 OR 25
27	7 AND 26
28	27 [DT 2016-2020]

Medline: Healthcare Databases Advanced Search

**Table:**

	Search term
1	(serious game*).ti,ab
2	(video game*).ti,ab
3	‘VIDEO GAMES’/
4	2 OR 3
5	(therap*).ti,ab
6	4 AND 5
7	1 OR 6
8	(mental health).ti,ab
9	‘MENTAL HEALTH’/
10	(mental illness).ti,ab
11	(depression).ti,ab
12	‘DEPRESSION’/
13	(anxiety).ti,ab
14	exp ‘ANXIETY DISORDERS’/
15	(problem drinking).ti,ab
16	(schizophrenia).ti,ab
17	SCHIZOPHRENIA/
18	(‘obsessive compulsive disorder’ OR ‘OCD’).ti,ab
19	‘OBSESSIVE-COMPULSIVE DISORDER’/
20	(‘post-traumatic stress disorder’ OR ‘PTSD’).ti,ab
21	‘STRESS DISORDERS, POST-TRAUMATIC’/
22	(‘attention-deficit hyperactivity disorder’ OR ‘ADHD’).ti,ab
23	‘ATTENTION DEFICIT AND DISRUPTIVE BEHAVIOR DISORDERS’/ OR ‘ATTENTION DEFICIT DISORDER WITH HYPERACTIVITY’/
24	8 OR 9 OR 10 OR 11 OR 12 OR 13 OR 14 OR 15 OR 16 OR 17 OR 18 OR 19 OR 20 OR 21 OR 22 OR 23
25	7 AND 24
26	25 [DT 2016-2020]
